# Oxidative stress induces coordinated remodeling of RNA-enzyme interactions

**DOI:** 10.1016/j.isci.2021.102753

**Published:** 2021-06-19

**Authors:** Ana M. Matia-González, Ibtissam Jabre, Emma E. Laing, André P. Gerber

**Affiliations:** 1 Department of Microbial Sciences, School of Biosciences and Medicine, Faculty of Health and Medical Sciences, University of Surrey, Guildford, Surrey, GU2 7XH, UK; 2 Department of Biochemistry and Molecular Biology I, Faculty of Sciences, University of Granada, Avda Fuentenueva s/n, Granada 18071, Spain

**Keywords:** molecular network, microbial metabolism, proteomics

## Abstract

RNA-binding proteins (RBPs) are key post-transcriptional regulators that play a substantial role during stress adaptation. Recent proteome-wide surveys have uncovered a large number of new and “unconventional” RBPs such as metabolic enzymes, yet little is known about the reconfiguration of the RNA-binding proteome (RBPome) and RNA-enzyme interactions in response to cellular stress. Here, we applied RNA-interactome capture to monitor the dynamics of the mRBPome upon mild oxidative stress in the yeast *Saccharomyces cerevisiae*. Among the 257 proteins that significantly changed RNA associations, we observed the coordinated remodeling of RNA-binding enzymes — particularly of the central carbon metabolism — that complemented known metabolic responses. Furthermore, we recognized the propensity for paralogous specific alterations of enzyme-RNA interactions. Our results suggest coordinated cross talk between RNA-enzyme interactions and intermediary metabolism to maintain the physiological and molecular balance upon oxidative stress, perhaps through specialization of paralogous during evolution.

## Highlights

• Oxidative stress induces the rearrangement of 257 proteins on polyadenylated RNAs
• Coordinated response of RNA-enzyme interactions and metabolism
• Yeast RNA-binding enzymes are paralog specific
• Integration of three different mass spectrometry analysis tools

## Graphical abstract

**Figure:**
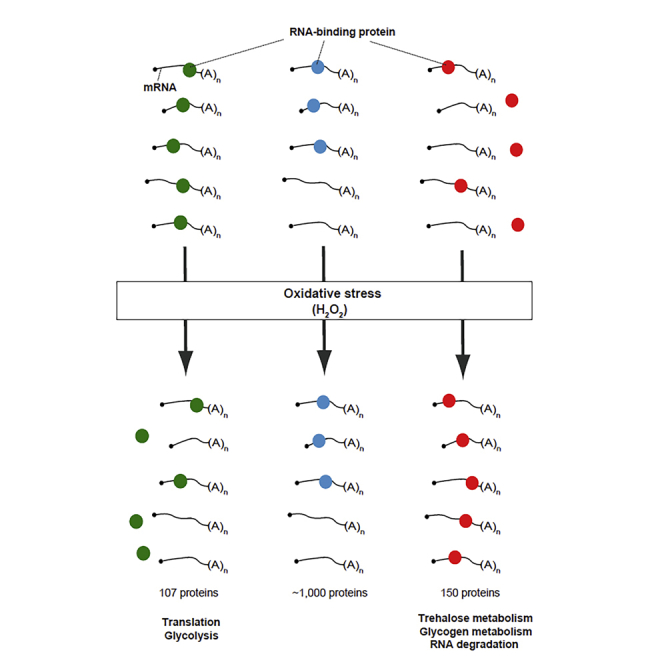

## Introduction

RNA-binding proteins (RBPs) play essential roles in regulating the transcription, processing, transport, translation, and turnover of RNAs (Gebauer et al., 2020; Glisovic et al., 2008). Recent deployment of proteome-wide assays have systematically identified sets of proteins that interact with RNAs across different organisms and cellular systems, referred to as the RNA-binding proteome (RBPome), revealing novel and “unconventional” RBPs such as metabolic enzymes (reviewed in Albihlal and Gerber, 2018; Hentze et al., 2018). Nevertheless, there is still little understanding as to how the RBPome reconfigures under changing environmental conditions or during development. This is a critical gap given that stress-induced reconfiguration of RNA-protein interactions is associated with essential post-transcriptional processes such as RNA degradation and translation, founding a premise for cellular stress adaptation. Indeed, apoptotic stress in L4-staged larvae of the nematode *Caenorhabditis elegans* induced changes in approximately 16% of the mRNA binding proteome (mRBPome), including translation initiation and elongation factors (Matia-Gonzalez et al., 2015), and arsenite-induced oxidative stress in mammalian cells affected ∼6% of all RNA-protein associations regulating translation and RNA degradation (Backlund et al., 2020). In the yeast *Saccharomyces cerevisiae*, applying total RNA-associated proteome purification (TRAPP), acidic stress induced remodeling of 10% of RNA-associated proteins involved in ribosome biogenesis, translation and the formation of P-bodies and stress granules (Shchepachev et al., 2019), while heat shock and glucose depletion primarily reduced RNA associations of translation factors, indicating common mechanisms of translational repression (Bresson et al., 2020).

Oxidative stress occurs as a consequence of aerobic respiration. Reactive oxygen species (ROS) are produced primarily through leakage of electrons from the mitochondrial electron transport chain and thus cells must uphold an antioxidant system to maintain their redox balance (Schieber and Chandel, 2014). Oxidative stress occurs when the cellular scavenging mechanisms are unable to cope with existing ROS, resulting in the damage of proteins, lipids, carbohydrates, and nucleic acids through oxidation (Morano et al., 2012; Schieber and Chandel, 2014). Yeast cells are exposed to oxidative stress during freezing and drying processes, which is of particular concern to the brewing and baking industries; whilst in humans, oxidative damage is associated with a number of pathologies including cancer, atherosclerosis, diabetes, arthritis, as well as neurodegenerative and age-related diseases (Pizzino et al., 2017; Schieber and Chandel, 2014).

Oxidative stress leads to activation of evolutionary conserved signaling pathways, that can either activate transcription factors that in turn promote stress-specific transcriptional responses, or modulate protein synthesis and RNA stability through modification of translation factors or the activity of specific RBPs (Blevins et al., 2019; Gerashchenko et al., 2012; Grant, 2011; Kershaw et al., 2015; Morano et al., 2012; Rowe et al., 2014). Furthermore, oxidative stress triggers a rapid metabolic response by rewiring the carbon metabolism, where glycolytic flux is diverted into the pentose phosphate pathway (PPP) (Christodoulou et al., 2019; Ralser et al., 2007); and the production of reserve polysaccharides such as glycerol and trehalose (Parrou et al., 1997). The oxidative branch of the PPP produces NADPH required for the regeneration of reduced glutathione, a major ROS scavenger in cells (Wood, 1986). Interestingly, many of the enzymes involved in central carbon metabolism have been identified as unconventional RBPs in diverse species (Beckmann et al., 2015; Marondedze et al., 2016; Matia-Gonzalez et al., 2015; reviewed in Albihlal and Gerber, 2018; Hentze et al., 2018). However, the relationship between RNA-enzyme interactions and metabolic processes remains elusive.

Here we applied RNA-interactome capture (RIC) to profile oxidative stress induced changes of the mRBPome in the yeast *Saccharomyces cerevisiae*. Surprisingly, we detected a strong rewiring of metabolic enzyme-RNA associations, a substantial fraction of them relating to enzymes acting in carbon and amino acids metabolism. Additionally, we observed that oxidative stress induced re-arrangements of enzyme-RNA interactions are highly paralogous-specific, suggesting evolutionary specification. These results suggest the coordinated response between enzyme-RNA interactions and metabolic activity for adaptation to cellular stress.

## Results

### Identification of mRNA binding proteins upon hydrogen peroxide induced oxidative stress in yeast

**Figure 1:**
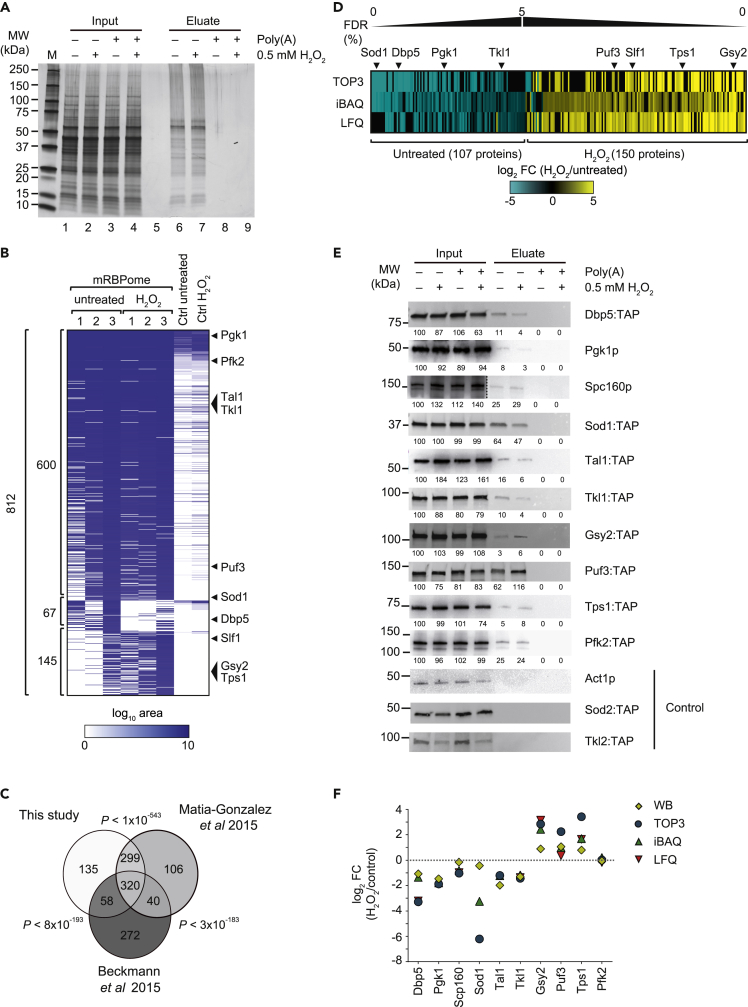
Identification of RBPs in *S. cerevisiae* upon H_2_O_2_ treatment (A) Silver stained polyacrylamide gel. Lanes 1–4 correspond to 0.05% of the input extracts; lanes 6–9 to 10% of eluates containing proteins UV-crosslinked to poly(A) mRNA. Excess competitor polyadenylic acids added to control extracts prior to mRNA isolation served as a negative control and was designated as Poly(A). A marker (MW) with molecular weights in kDa is indicated to the left. (B) Heatmap representing the abundance of 812 proteins comprising the yeast oxidative stress mRBPome identified with TOP3 analysis. Columns refer to three independent experiments with untreated and H_2_O_2_ treated cells, and the averaged control samples. Rows represent individual proteins. The white-blue color bar represents log_10_ transformed raw (non-normalized) MS peak areas of respective proteins. 600 proteins selected in both control and H_2_O_2_ treated cells, 67 proteins selected in untreated samples and 145 proteins in stressed cells have been grouped. (C) Venn diagram showing overlap of the mRBPome defined in this study as compared to previous studies (Beckmann et al., 2015; Matia-Gonzalez et al., 2015). The p value (hypergeometric test) relates to the significance of the overlap. (D) Heatmap depicting relative changes of 257 proteins in the mRBPome in H_2_O_2_ treated cells (FDR ≤5%). Columns refer to proteins, rows to the MS quantification method. Log_2_ fold-changes (H_2_O_2_/untreated) are indicated with the blue-yellow color bar. (E) Western Blot (WB) monitoring differential poly(A) RNA association of indicated proteins. Act1, Sod2, and Tkl2 are non-RNA binding controls. TAP refers to the detection of tandem affinity purification-tagged proteins. Poly(A) designates the addition of excess competitor poly(A). Quantified signals relative to untreated input samples (=100) are displayed below each lane. (F) Graph displaying log_2_ FC of RNA-protein interactions as determined by the three MS quantification methods (TOP3, iBAC, LFQ) and WB for the indicated proteins.

To investigate the dynamics of the mRBPome upon stress, we monitored the changes in yeast cells upon application of a short and mild oxidative stress. *S. cerevisiae* cells were grown to mid-log phase in rich glucose-containing medium (YPD), and oxidative stress was induced by addition of 0.5 mM hydrogen peroxide (H_2_O_2_) for 15 min. We recorded increased expression of *HSP30* and *GRE2* mRNAs, both known to be induced by sub-lethal doses of H_2_O_2_ (Alejandro-Osorio et al., 2009; Tsang et al., 2014) (Figure S1A). We then performed RIC on H_2_O_2_ stressed and untreated control cells STAR Methods (Matia-Gonzalez et al., 2015). In brief, proteins were covalently crosslinked to nucleic acids *in vivo* by UV irradiation of cells, and poly(A)-containing RNAs with bound proteins were purified from cell lysates using oligo[dT]_25_ beads. We wish to note that the isolation of poly(A) RNA is thought to mainly recover mRNA-interacting proteins, although other types of RNA bearing a poly(A) tail could also be recovered. As a negative control, competition experiments with exogenously added polyadenylic acids were performed for each experiment (Matia-Gonzalez et al., 2015). The integrity of total RNA isolated from crosslinked and stress-treated cells (Figure S1B), as well as the selective enrichment of proteins in eluates compared to input extracts, was assessed with a silver-stained gel (Figure 1A).

Three independent experiments (biological replicates) and respective control samples to demarcate non-specific binders were subjected to liquid chromatography-tandem mass spectrometry (LC-MS/MS). To enable robust data interpretation and minimize method specific detection bias, we applied three label-free MS quantification approaches, namely TOP3 (Silva et al., 2006) which we previously used to determine the yeast mRBPome (Matia-Gonzalez et al., 2015), intensity-based absolute quantification (iBAQ) and label-free quantification (LFQ) implemented in MaxQuant (Cox et al., 2014; Cox and Mann, 2008). Using TOP3, we identified 812 proteins in at least two of three replicate experiments from untreated or stressed cells with a false discovery rate (FDR) of less than 1% and represented by at least two different peptides (Figure 1B; data are given in Table S1A). Among those, 135 were not detected in previous yeast oligo[dT]-based RIC studies — including our own TOP3-based analysis (Beckmann et al., 2015; Matia-Gonzalez et al., 2015; Mitchell et al., 2013) — and hence, those proteins may constitute novel RBPs (Figure 1C). Applying the same selection criteria, we retrieved 1,276 and 933 proteins with iBAQ and LFQ modes, respectively (data for all MS analysis are given in Table S1A-C). Noteworthy, while data correlated well among biological replicates (Pearson correlation, *r* = 0.7–0.85) (Figure S2A) and fairly well among the different quantification methods (Pearson correlation, *r* = 0.57–0.73) (Figure S2B), the fold-changes (FC) were generally greater with the TOP3 approach as compared with iBAQ or LFQ (stdev FC: 1.839 (TOP3), 0.9748 (iBAQ) and 1.223 (LFQ)).

To determine a list of proteins that significantly changed RNA-associations, we selected 251, 408, and 307 proteins that changed their mRNA associations with FDR ≤5% upon H_2_O_2_ stress with the TOP3, iBAQ, and LFQ analysis approach, respectively (Figure S2C, Table S1D). To account for the deviation in the selected list of proteins, we further considered all of the proteins that were selected by at least two different analyses, rendering 257 proteins or ∼25% of all identified proteins (Table S1E). Among those, 107 proteins became less, and 150 proteins increasingly associated with poly(A) RNA in H_2_O_2_ treated cells (Figure 1D). Of note, the latter group included the RBPs Slf1 and Puf3 that have reported roles in modulating translation of mRNAs during the oxidative stress response (Kershaw et al., 2015; Rowe et al., 2014 #12).

We further validated our data by immunoblot analysis for several of the identified proteins, including the RBPs Dbp5, Puf3, and Scp160, as well as the metabolic enzymes Gsy2, Pgk1, Sod1, Tal1 (FDR_TOP3_ = 5.06), Tkl1, Tps1, and Pfk2. As negative controls, we included Act1, Sod2 and Tkl2, which have not been identified by MS and are therefore not expected to bind RNA (Figure 1E). The quantification of relative fold-changes measured by immunoblot analysis approximated the changes determined with the different MS quantitative methods (Figure 1F). Thereby, best consistency was seen with iBAQ mode generated MS data, while TOP3 tends to overestimate and LFQ underestimate the relative changes in RNA-protein associations. In conclusion, the implementation of three MS quantification methods, the detection of known RBPs involved in stress response, and the validation by immunoblot analysis strongly supports the reliability of the data obtained as a good measure for relative changes in poly(A) RNA-interacting proteins upon H_2_O_2_ stress.

### Alteration of the oxidative stress-induced mRBPome is not correlated with transcriptional/translational changes but connects to mRNA targets

To examine whether changes in RNA-protein associations could be due to underlying changes in respective mRNA levels, translation or protein levels, we further considered two published datasets reporting changes in transcriptome and translatome upon addition of 0.2–0.4 mM H_2_O_2_ for 5 to 30 min, similar conditions to those reported here (Alejandro-Osorio et al., 2009; Gerashchenko et al., 2012); as well as a data set reporting relative changes of the yeast proteome upon application of 1.5 mM H_2_O_2_ for one hour (Blevins et al., 2019). Interestingly, even at these relatively harsh conditions, only minor changes in protein levels (stdev = 0.32) were reported, suggesting that imposed changes in the proteome under our chosen mild oxidative stress conditions are negligible and generally lower than the observed variation across replicate MS measurements (average stdev ∼0.5). In any case, based on average FC measured across the three MS analysis tool (Figure S3A), no correlation was observed between our experimentally determined changes in poly(A) RNA-protein associations and changes in the respective mRNA levels (*r* = 0.03–0.08), the translational rate (*r* = 0.11–0.12), or protein levels (*r* = −0.08) (Figure S3B). Thus, we conclude that changes in the mRBPome could not be simply related to changes in corresponding mRNA, translation, and protein levels.

We further reasoned that altered poly(A) RNA associations of RBPs upon oxidative stress could relate to the different levels of corresponding mRNA targets. To address this, we searched for published targets of the 257 proteins that changed RNA-associations upon oxidative stress. RNA targets for 13 proteins, as previously determined by RBPs immunoprecipitation microarray analysis (RIP-Chip) were identified, namely Aco1, Gre3, Mdh1, Meu1, Nsr1, Scp160, Sti1, Pin4, Puf3, Puf5, She2, Slf1, and Ssd1 (Hogan et al., 2008; Schenk et al., 2012; Scherrer et al., 2010). As a control group, we selected RNA targets for another ten published RBPs that did not significantly change RNA association upon oxidative stress and yet have similar characteristics to the previous group, namely two metabolic enzymes Idh1 and Tdh3; members of the Puf-family proteins Puf1p, Puf2p, Puf4p and other RBPs like Gbp2, Mex67, Nab3, Pab1, and Sro9 (Hieronymus and Silver, 2003; Hogan et al., 2008; Schenk et al., 2012; Scherrer et al., 2010). We referenced those on the imposed changes in RNA levels upon application of 0.4 mM H_2_O_2_ for 20 min (Alejandro-Osorio et al., 2009). Interestingly, we observed that the expression levels of RNA targets for 8 of the selected 13 oxidative stress responding RBPs (62%; Aco1, Nsr1, Sti1, Pin4, Puf3, Puf5, Slf1, and Ssd1) were significantly altered upon oxidative stress, but only for 3 of the 10 RBPs in the control group (∼30%; Puf4, Mex67, and Sro9, a paralogue of Slf1 with overlapping mRNA targets) (Figure S3C). Thus, it seems that proteins that significantly changed RNA-associations upon mild oxidative stress show strong disposition for imposed changes in their mRNA target levels. For example, Puf3p mRNA targets, which mainly code for nuclear encoded mitochondrial proteins, including structural components of the ribosome, tRNA ligases, translational regulators, and other proteins involved in the mitochondrial organization and biogenesis (Gerber et al., 2004; Hogan et al., 2008), are significantly increased upon oxidative stress (Riordan et al., 2011) and along with increased association of Puf3p with poly(A) RNAs, which is in line with the previously reported role for Puf3p acting as a translational repressor during oxidative stress (Rowe et al., 2014). Conversely, the mRNA targets for Aco1p, which comprise mitochondrial mRNAs encoding proteins involved in aerobic respiration (e.g. oxidative phosphorylation, electron transport (Hogan et al., 2008) are significantly less abundant upon oxidative stress (Alejandro-Osorio et al., 2009), which goes along with reduced association of Aco1p with poly(A) RNAs upon oxidative stress. While the function of Aco1p in mRNA metabolism is not known, the reduction of Aco1's mRNA association and mRNA target levels could lead to the speculation about potential roles in controlling the stability of mitochondrial encoded mRNAs upon oxidative stress.

### Coordinated response of RNA-enzyme interactions and metabolism upon oxidative stress

**Figure 2:**
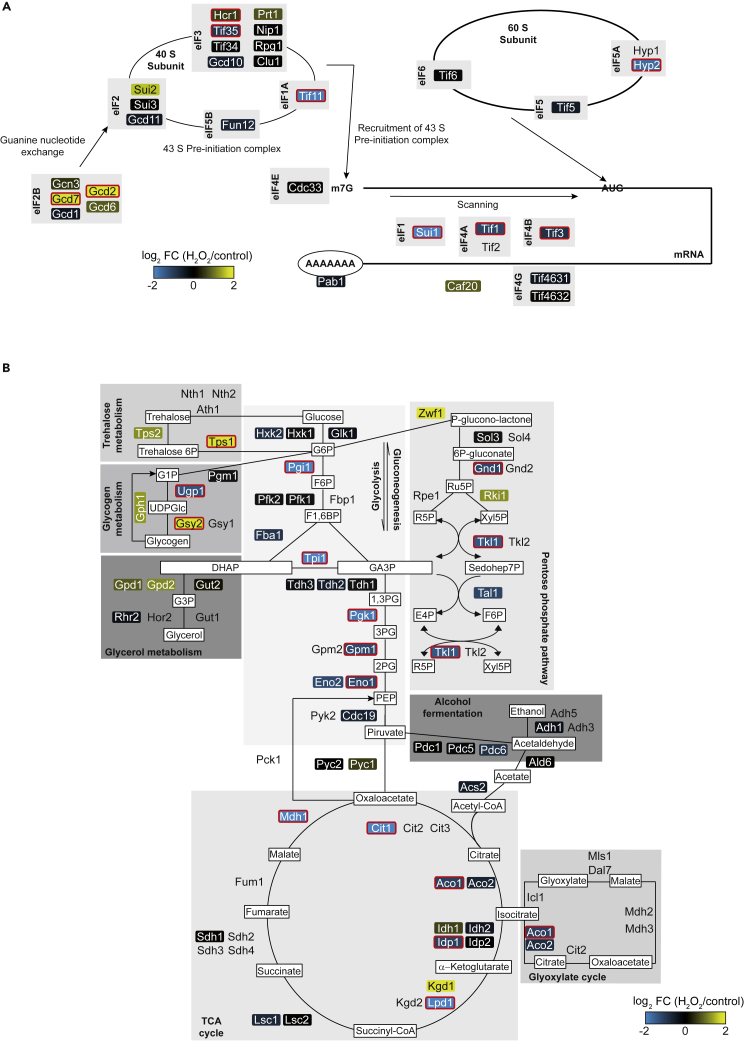
Changes in poly(A) RNA associations for translational initiation factors and enzymes of carbon metabolism upon mild oxidative stress (A) Schematic view of translation initiation. Average log_2_ FC for depicted proteins are indicated with the blue-black-yellow color gradient; proteins with significant differential association (FDR ≤5%) are highlighted with a thick red border. (B) Central carbon metabolism. The color gradient and significant differential associations of proteins are specified as in (A). Metabolites are depicted in white boxes: G6P, glucose 6-phosphate; F6P, fructose 6-phosphate; F1, 6BP, fructose 1,6-bisphosphate; DHAP, dihydroxyacetone phosphate; GA3P, glyceraldehyde 3-phosphate; 1,3PG, 1,3-bisphosphoglycerate; 3PG, 3-phosphoglycerate; 2PG, 2-phosphoglycerate; PEP, phosphoenolpyruvate; Ru5P, ribulose 5-phosphate; R5P, ribose 5-phosphate; Xyl5P, xylulose 5-phosphate; Sehohep7P, sedoheptulose 7-phosphate; E4P, erythrose 4-phosphate; G1P, glucose 1-phosphate; UDPGlc, UDP-glucose; G3P, glycerol 3-phosphate.

To unravel common features among the 257 selected proteins that exhibited differential RNA association upon stress, we searched for overrepresented Gene Ontology (GO) terms and pathways (Table 1, the complete analysis is provided in Tables S2A and S2B). Overall, a substantial fraction of these proteins (total 74 proteins) are annotated as “RNA-binding” (p < 1 × 10^−14^), which includes the stress-dependent RBPs Puf3 and Slf1. More specifically, the 150 proteins increasingly associated with poly(A) RNA upon oxidative stress have assigned functions in RNA localization, ribosome biogenesis, RNA processing, and RNA degradation. Interestingly, the latter includes one of the two TRAMP complexes (Trf4/Pap2, Air1, Mtr4; p < 2 × 10^−4^), an evolutionary conserved complex involved in RNA surveillance and quality control (Delan-Forino et al., 2020; Schmid and Jensen, 2019). Possibly, the increased association with RNA processing and RNA degradation pathways upon oxidative stress could relate to selective removal of damaged RNAs produced through oxidation (Li et al., 2006). Conversely, a significant fraction of translation initiation factors showed significantly reduced association with RNA upon oxidative stress (total 9 proteins, p < 4.3 × 10^−4^), which aligns with the commonly observed ceasing of translation initiation upon stress (Shenton et al., 2006) (Figure 2A). Nonetheless, two components of eukaryotic initiation factor 2B complex (eIF2B), Gcd2 and Gcd7 showed increased RNA association upon stress. While direct RNA-binding of these guanyl-nucleotide exchange factors has not been reported previously, we speculate that the enhanced RNA associations may relate to the formation of eIF2B bodies upon oxidative stress similar to that under severe glucose deprivation stress (Moon and Parker, 2018).

**Table 1:** Enriched functional themes among proteins that are less or more associated with poly(A) RNAs in H_2_O_2_ treated cells

GO^a^/KEGG^b^/WP^c^ term	# Proteins	p value	Protein set
Biosynthesis of amino acids^c^	20	3.6 × 10^−10^	Less associated with poly(A) RNA (107 proteins)
Translational initiation^a^	9	4.3 × 10^−4^	
Principle pathways of carbon metabolism^c^	12	1.7 × 10^−4^	
Ribosome biogenesis^a^	29	6.7 × 10^−6^	More associated with poly(A) RNA (150 proteins)
RNA processing^a^	35	1.8 × 10^−5^	
RNA localization^a^	19	2.9 × 10^−5^	
RNA degradation^b^	8	1.8 × 10^−3^	

a Gene Ontology (GO).

b KEGG analysis.

c WikiPathway (WP).

Besides changes of known RBPs acting in post-transcriptional gene regulation, we were particularly intrigued by the apparent coordinated response among metabolic enzymes. Overall, 64 proteins are associated with “metabolic pathways” (KEGG) and therefore slightly overrepresented among the 257 proteins changing RNA associations significantly upon H_2_O_2_ treatment (p < 0.015). Most striking, 12 enzymes acting in “principle pathways of carbon metabolism” were enriched among the 107 proteins with decreased RNA association upon oxidative stress (Table 1). This attracted our interest since carbon metabolism is highly responsive to oxidative stress (Mullarky and Cantley, 2015; Ralser et al., 2007). Glycolysis is generally repressed and rerouted to the oxidative branch of the PPP for production of NADPH to adjust the redox balance in cells (Mullarky and Cantley, 2015), the glycerol metabolism for production of triglycerides (Ralser et al., 2007); and toward the production of trehalose, a protector against oxidative stress (Mizunoe et al., 2018). Analogous to these changes in intermediary metabolism, we found that enzymes of the glycolytic pathway (Pgi1, Tpi1, Pgk1, Gpm1 and Eno1) and downstream tricarboxylic acid (TCA) cycle (Cit1, Aco1, Lpd1, Mdh1), as well as components of the PPP (e.g., Gnd1, Tal1 (FDR_TOP3_ = 5.06), Tkl1) commonly exhibited significantly lowered RNA associations in H_2_O_2_ treated cells (Figure 2B). Likewise, gene set enrichment analysis (GSEA) across the changes implemented on the entire mRBPome (irrespective of any cut-off) further confirmed the significant reduction of RNA-associations for glycolytic enzymes (KEGG/WikiPathway; p = 0) (complete GSEA is provided in Table S2C). Conversely, Gsy2p and Tps1p, both involved in the synthesis of glycerol and trehalose, respectively, showed increased poly(A) RNA association upon H_2_O_2_ treatment, in line with the activation of glycogen and trehalose production upon oxidative stress (Mizunoe et al., 2018; Ralser et al., 2007) (Figure 2B). Hence, alterations in RNA binding activity of enzymes acting in glycolysis and associated glycogen and trehalose pathways generally correlate with the imposed changes in metabolic flux, suggesting a coordinated response between RNA-associations and metabolic activity.

Besides major effects on enzymes acting in central carbon metabolisms, we also found altered RNA associations among enzymes acting in nucleotide (e.g. Guk1, Gua10, Ade1, Ade17, Hpt1, Ado1), fatty acids (e.g. Frm2) and amino acid metabolism. Regarding the latter, 20 enzymes associated with the “biosynthesis of amino acids” (KEGG) were found among the set of proteins with significantly decreased mRNA association upon oxidative stress. Likewise, GSEA across the entire mRBPome revealed significantly reduced RNA associations for proteins allocated to the “biosynthesis of amino acids” (KEGG, p < 0.0012). This includes proteins required for the synthesis of aspartate (Aat2), asparagine (Asn1, Asn2), glutamate (Gdh1, Glt1), serine/lysine (Hom2, Hom6, Lys4), methionine (Met6), cysteine (Sah1), threonine (Thr4), isoleucine (Bat1, Mmf1), and others. The relevance of the herein observed changes in RNA-enzyme interactions is not known but may suggest the existence of an RNA-imposed regulatory layer for amino acids synthesis during stress.

### Oxidative stress alters mRNA associations of paralogous enzymes

We next asked whether functional specification toward RNA-binding enzymes could have been implemented through specialization of paralogous genes after early genome duplication in yeast. We therefore considered a list of 1,094 proteins, representing 547 paralogous pairs (Byrne and Wolfe, 2005). One hundred forty four of the 812 yeast mRBPome (TOP3 defined) proteins have an annotated paralog. Of these, there were 53 paralogous pairs (total 106 proteins) that were both detected to interact with RNA, while 91 proteins were identified irrespective of its paired paralog, suggesting possible specialization toward RNA binding (Table S3). For example, only one paralog for enzymes acting in the central carbon metabolism enzymes, such as phosphoglycerate mutase (Gpm1), 6-phosphogluconolactonase (Sol3), 6-phosphogluconate dehydrogenase (Gnd1), transketolase 1 (Tkl1), and glycogen synthase (Gsy2) were identified as RNA-binders (Figure 2B). Generally, we could not detect an obvious bias for RNA-binding versus the non-RNA binding paralogous in regard of their protein localization (Breker et al., 2014). However, we found that the 91 RNA-binding paralogous are significantly higher expressed compared to their non-RNA binding paralogous (Mann-Whitney test, p < 0.0001), considering a unified protein abundance data set for yeast cells (Ho et al., 2018). While this could suggest missed detection of low abundant paralogous proteins by MS, we reproducibly identified likewise low abundant RBPs in the mRBPome by MS. In particular, while 12 non-RNA binding paralogs are low abundant (up to 2,000 copies per cell), 15 likewise low abundant RBPs were reproducibly identified by MS, showing that MS is not detrimental for detection of low abundant proteins in the mRBPome.

Interestingly although, among the 257 proteins that changed RNA associations significantly upon H_2_O_2_ treatment stress, we found only two paralogous pairs (Asn1/Asn2, Ptc2/Ptc3; both pairs increasing RNA-associations upon oxidative stress) but 44 proteins that had a non-RNA binding paralog. In this regard, immunoblot analysis with tagged strains confirmed that Tkl1 but not Tkl2, and cytosolic superoxide dismutase 1 (Sod1) but not its mitochondrial paralog Sod2 interacts with poly(A) RNA, respectively (Figure 1E). Furthermore, enzymes acting in amino acid biosynthesis (6 proteins, p < 10^−5^) and carbon metabolism (12 proteins, p < 0.2 × 10^−4^) are overrepresented among those 44 unique paralogues as compared with all annotated paralogous proteins in yeast. Thus, oxidative stress may preferentially alter mRNA associations of selected paralogues acting in metabolic processes. If so, we speculate that environmental stress could have been a driver for specialization of RNA-binding paralogues during evolution.

## Discussion

We investigated the plasticity of the yeast mRBPome upon exposure of yeast cells to short mild oxidative stress caused by exposure to H_2_O_2_. By performing RIC and integrating three complementary MS analysis methods, we defined a list of 257 proteins — corresponding to 25% of the yeast mRBPome — that changed association with poly(A) during oxidative stress (FDR <5%). This included stress-regulated RBPs with known roles in translation and RNA degradation, reminiscent of previous observations made with arsenite-treated human cells, which promotes a strong oxidative stress response (Backlund et al., 2020). Likewise connections to translation and RNA decay have also been previously made in yeast by unraveling changes of the total RBPome upon acidic stress, heat shock, and in glucose deprived cells (Bresson et al., 2020; Shchepachev et al., 2019). Importantly though, our study was focused on poly(A) interacting RNAs, which mainly comprises mRNAs, while the latter studies applied the TRAPP technique to assess changes of all RBPs, irrespective of its RNA target group. Nevertheless, we found a significant overlap of the identified RBPs: for instance, 309 proteins of our herein determined mRBPome (812 protein determined with TOP3) were also identified by Bresson et al. (2020) under glucose starvation and heat shock conditions (total 711 proteins) (p < 1.1 × 10^−114^, hypergeometric test). More surprising though, we observed profound changes in RNA-enzyme interactions, many of them acting in carbon and amino acids metabolisms that commensurate with altered metabolisms. Thereby, the imposed reconfiguration of enzymes-RNA interaction appeared to be paralogue-specific, proposing stress adaptation as a driver for the specialization of RNA-binding enzymes during evolution.

Oxidative stress leads to the formation of ROS damaging biomolecules and is detrimental to cellular redox balance. As a reaction, ROS imposes rapid repression of the glycolytic flux, which triggers the rerouting of intermediate metabolites to associated pathways to adjust cellular redox balance (Christodoulou et al., 2019; Mullarky and Cantley, 2015). Interestingly, we found that the stress-induced mRBPome showed a compromise in binding activity toward redox-maintaining metabolic pathways, as evident from a significant repression of the RNA-binding of several glycolytic enzymes. These include Tpi1p, which plays a key role in the oxidative stress response (Ralser et al., 2007); several enzymes of the downstream TCA cycle, which goes along with the compromised mitochondrial activity imposed through oxidative stress induced damage (Kowaltowski and Vercesi, 1999); and the activation of Gsy2 and Tps1, required for glycogen and trehalose production with imminent roles in stress response (Arguelles, 2000; Benaroudj et al., 2001; Xu and Tsurugi, 2006). These obvious paralleled responses between RNA-binding and metabolic activity could suggest the presence of previously overlooked sensors and control units that directly link availability of metabolites with post-transcriptional control through enzyme-bound mRNAs. Importantly, the herein proposed coordination is likely not restricted to central carbon metabolisms, but expands to the entire metabolic landscape, namely for synthesis of amino acids, nucleotides or fatty acids to maintain a balanced physiological state upon oxidative stress. For example, Frm2p, which plays an essential role in the repression of genes encoding proteins acting in fatty acid biosynthesis upon oxidative stress (de Oliveira et al., 2010), showed the most increased poly(A) associations upon oxidative stress among all recorded proteins. However, we wish to note that directly correlated changes in RNA-binding and metabolic/enzymatic activity are not necessarily the rule as seen for components of oxidative (i.e. Gnd1) and non-oxidative branch of the PPP. In regard to the latter, the substrate and product (sedohep7P) for Tkl1 and Tal1, respectively, gets rapidly accumulated in H_2_O_2_ stressed yeast cells (Ralser et al., 2009), while we measured decreased poly(A) RNA associations for these enzymes, indicating the RNA binding could impact on enzymatic reaction kinetics.

We also recognized that RNA-binding enzymes often refer to selected paralogs. Interestingly, likewise analysis of the recent RBPome data from Bresson et al. (Bresson et al., 2020) revealed 24 paralog pairs and 67 proteins interacting with RNA irrespective of its paralogous pair, further supporting specification of RNA-binding paralogous proteins. The pressure to adapt to diverse environmental stress could have been a major driver toward such functional specification (Conant and Wolfe, 2008), as seen by the high proportion of enzyme paralogous that significantly changed RNA associations upon oxidative stress (albeit paralog specific stress-adaption is also seen among canonical RBP complexes, as observed for the TRAMP complex). While one paralog could implement metabolic activity, the paired RNA-binding paralog may have further evolved to undertake additional functions in RNA metabolism (although RNA-binding activity could also have been lost during evolution). For example, we found strongly diminished RNA-binding of cytosolic Sod1p upon oxidative stress, while mitochondrial Sod2 was not found to interact with RNA (Figure 1E). In agreement with this finding, it has been reported that Sod1p is highly responsive to oxidative stress and translocates to the nucleus for transcriptional activation of stress-related genes such as *YAP1* (Tsang et al., 2014). Thus, poly(A) RNA could act as a scaffold to anchor Sod1p in the cytoplasm, which is released upon oxidative stress, enabling the Sod1p translocation to the nucleus — a hypothesis to be tested in the future. Thus, whilst a few examples for specialized paralogous RNA-binding enzymes have been described (Albihlal and Gerber, 2018; Hentze et al., 2018), our data suggest that functional specialization among paralogous could be far more common than previously thought.

In conclusion, our study suggests a coordinated response between RNA-enzyme interactions and intermediary metabolism to maintain the physiological and molecular balance upon oxidative stress, and we speculate that this coordination could have in part be promoted through specialization of paralogous proteins. It will be interesting to see whether likewise associations can be made under different stress conditions, and whether the correspondence with metabolic potential remains. If this is the case, monitoring of RBPome dynamics could become a new observatory tool to monitor metabolic and other diseases.

### Limitations of the study

The chosen method (RIC) to characterize the mRBPome upon oxidative stress was based on capturing poly(A) RNAs from cells, a major component of it comprising mRNAs; likely disregarding changes for RBPs that preferentially interact with other RNA species, such as rRNAs and tRNAs. We have also analyzed changes of the mRBPome under one mild stress conditions. Therefore, we do not know whether the observed coherent changes in RNA-enzyme interactions are specific to the chosen stress conditions, and in how far they are dependent on the concentration and duration of stress application with H_2_O_2_. The comparison of our experimentally determined mRBPome to inferred changes of the transcriptome and proteome was based on data from published studies, which may provide a good approximation but may not fully reflect changes seen under our particularly chosen conditions. Finally, our study indicates a coordinated response between RNA-enzyme interactions and intermediary metabolism; however specific key targets and mechanism remain elusive. Further studies are required to provide mechanistic details and consolidate the observed connections between metabolism and RNA binding.

## STAR★Methods

### Key resources table

**Table:** 

REAGENT or RESOURCE	SOURCE	IDENTIFIER
**Antibodies**		

Rabbit anti-Scp160	Laboratory of M. Seedorf	https://doi.org/10.1074/jbc.M009430200
Mouse anti-Act1	MP Biomedicals	Cat # 08691002; RRID:AB_2335304
Rabbit anti-Pfk	Laboratory of J. Heinisch	https://doi.org/10.1074/jbc.m007131200
Rabbit anti-Zwf1	Sigma	Cat # A9521; RRID:AB_258454
Mouse anti-Pgk1 - clone 22C5D8	AbCam	Cat # ab113687; RRID:AB_10861977
PAP reagent	Sigma	Cat #P1291; RRID:AB_1079562
HRP-conjugated donkey anti-rabbit IgG	Amersham	Cat # NA9340; RRID:AB_772191
HRP-conjugated sheep anti-mouse IgG	Amersham	Cat # NA931; RRID:AB_772210

**Chemicals, peptides, and recombinant proteins**		

cOmplete EDTA-free protease inhibitors	Roche	Cat # 11873580001
		
DNase I	Promega	Cat #M6101
RNasIN Ribonuclease inhibitor	Promega	Cat #N2511
Poly(A)	Sigma	Cat #P9403

**Critical commercial assays**		

ZR RNA MiniPrep kit	Zymo Research	Cat #R1065
Dynabeads™ mRNA DIRECT™ purification kit	Life Technologies	Cat # 61,011
Microcon-10 kD Centrifugal filter unit with Ultracel-10 membrane	Millipore	Cat # MRCPRT010
4–12% NuPAGE Novex acrylamide gel	Thermo Scientific	Cat # NP0321BOX
Polyvinylidene difluoride (PVDF) membranes	Thermo Scientific Pierce	Cat # 88,518
Transcriptor high Fidelity cDNA synthesis kit	Roche	Cat # 05091284001

**Deposited data**		

Mass spectrometry data: RBPome in untreated and hydrogen peroxide treated yeast cells.	This paper	Table S1. PRIDE: PXD005943

Experimental models: Organisms/strains		
Saccharomyces cerevisiae strain BY4741: *MAT*a his3Δ1 leu2Δ0 met15Δ0 ura3Δ0	Euroscarf collection	Cat #Y00000

**Oligonucleotides**		

HSP30_Fwd, 5′-CTAGAGGGTTCAATGCACTTAT-3′	This paper	NA
HSP30_Rev 5′-CTCACCGTCTGGTTGAATAC-3′	This paper	NA
GRE2_Fw, 5′-CCGGAACTATTTGGTGGATAC-3′	This paper	NA
GRE2_Rev, 5-CCTCCGATACGATTAGTCTTTG-3′	This paper	NA
ACT1_Fw, 5′-GTCTGGATTGGTGGTTCTATC-3′	This paper	NA
ACT1_Rev, 5′-GGACCACTTTCGTCGTATTC-3′	This paper	NA

**Software and algorithms**		

Prism 8	GraphPad	https://www.graphpad.com/
GO term Finder v0.86		https://yeastgenome.org/goTermFinder
Webgstalt	Liao et al. (2019)	http://www.webgestalt.org/
Proteome DiscovererTM software v1.2	Thermo Scientific	
MaxQuant v1.6.1.0	Cox and Mann. (2008)	https://www.maxquant.org/
R v3.4.0	R Core Team (2017). R: A language and environment for statistical computing. R Foundation for Statistical Computing, Vienna, Austria.	http://www.R-project.org/
ImageJ Macro Language Programmer v1.46d	ImageJ, Fiji Wiki, and ImageJ documentation Wiki	ImageJ website: http://imagej.nih.gov/ij Fiji Wiki: http://pacific.mpi-cbg.de/wiki/index.php/Fiji ImageJ Documentation Wiki: http://imagejdocu.tudor.lu

### Resource availability

#### Lead contact

Further information and requests for resources and reagents should be directed to and will be fulfilled by the lead contact, André P. Gerber (a.gerber@surrey.ac.uk).

#### Materials availability

This study did not generate new materials.

#### Data and code availability

MS proteomics data are deposited at the ProteomeXchange Consortium (Perez-Riverol et al., 2019) via the PRIDE partner with dataset identifier PXD005943.

### Experimental model and subject details

All S. cerevisiae strains used in this study were derived from the BY4741 background strain (*MAT***a** *his3*Δ*1 leu2*Δ*0 met15*Δ*0 ura3*Δ*0*).

### Method details

#### Yeast cultures and oxidative stress induction

Strain BY4741 was grown in 500 mL YPD media (1% yeast extract, 2% peptone, 2% D-glucose) at 30°C with constant shaking at 220 r.p.m. To induce the oxidative stress response 0.5 mM H_2_O_2_ (Sigma, H1009) was added to the culture once cells reached mid-log phase (OD_600_ ∼ 0.6) and incubated for 15 min at 30°C. Cells were collected by centrifugation and washed in PBS. For UV-crosslinking, cells were resuspended in 25 mL of PBS and exposed to 1,200 mJ/cm^2^ of 254 nm UV light in a Stratalinker 1800 (Stratagene) with two 2-min breaks on ice and gentle mixing (Matia-Gonzalez et al., 2015). Cells were harvested by centrifugation and snap-frozen in liquid nitrogen.

#### *In vivo* capture of mRBPs in S. cerevisiae

RIC was essentially performed as described previously (Matia-Gonzalez et al., 2015). Pellet cells were resuspended in 4 mL lysis buffer (100 mM Tris-HCl, pH 7.5, 500 mM LiCl, 10 mM EDTA, 1% Triton X-100, 5 mM DTT, 20 U ml^−1^ DNase I (Promega, M6101), 100 U ml^−1^ RNasin (Promega, N2611), complete EDTA-free protease-inhibitor cocktail (Roche, 11836170001)) and mechanically broken with glass beads in a Tissue Lyser (RETSCH MM200, Qiagen) for 10 min at 30 Hz at 4°C. The cooled lysate was cleared by three sequential centrifugations at 4°C at 3,000 *g* for 3 min, and 5,000 *g* and 10,000 *g* for 5 min each. A negative control was introduced at this stage: the extract was supplemented with 20 mg of poly(A)(Sigma, P9403) for competition experiments. To capture polyadenylated RNAs for mRBPome analysis, one milligram of oligo[dT]_25_ Dynabeads (Life Technologies, 61,011) was equilibrated in lysis buffer, mixed with the extracts (∼5 mg) and incubated on a shaker for 10 min at room temperature (RT). The beads were collected with a magnet and the supernatant recovered for repeat incubations (see below). The beads were washed once with 500 μL of wash buffer A (10 mM Tris-HCl, pH 7.5, 600 mM LiCl, 1 mM EDTA, 0.1% Triton X-100) and twice with 500 μL wash buffer B (10 mM Tris-HCl, pH 7.5, 600 mM LiCl, 1 mM EDTA). The poly(A) RNA was eluted from beads in 60 μL of 10 mM Tris-HCl, pH 7.5 at 80°C for 2 min and collected. The entire procedure was repeated twice by reapplying the supernatant to the oligo[dT]_25_ beads, which were recovered after elution and washed three times with lysis buffer prior to reapplication. The three sequential eluates were combined and concentrated to 70 μL in a 0.5 mL Microcon-10 kD Centrifugal Filter Unit with Ultracel-10 membrane (Millipore, MRCPRT010). In total, we subjected 12 samples to MS analysis: three independent experiments (biological replicates) of the mRBPome in control conditions as well as three poly(A) competition control experiments; and three independent experiments for profiling the mRBPome under oxidative stress conditions along with three poly(A) competition experiments.

#### Total RNA extraction, cDNA synthesis and RT-PCR

Total RNA was isolated from 50 μL of extracts with the ZR RNA MiniPrep kit (Zymo Research, R1065) with in column DNA digestion. To monitor induction of *HSP30* and *GRE2* upon oxidative stress, reverse transcription (RT) was performed with 500 ng of total RNA combined with a mixture of with oligo[dT]_18_ and random hexamer primers, and the Transcriptor High Fidelity cDNA Synthesis Kit according to the manufactures' instructions (Roche, 05091284001). PCR was conducted with 1 μL (5%) of cDNA with the following primer pairs: HSP30_Fwd, 5′-CTAGAGGGTTCAATGCACTTAT-3′; HSP30_Rev 5′-CTCACCGTCTGGTTGAATAC-3′; GRE2_Fw, 5′-CCGGAACTATTTGGTGGATAC-3′; GRE2_Rev, 5-CCTCCGATACGATTAGTCTTTG-3′; ACT1_Fw, 5′-GTCTGGATTGGTGGTTCTATC-3′; and ACT1_Rev, 5′-GGACCACTTTCGTCGTATTC-3′. PCR was performed for 5 min at 94°C, 30 cycles at 94°C for 30 s, 57°C for 30 s, 72°C for 40 s, and 8 min at 72°C. PCR products were electrophoresed on a 2% agarose gel and visualized with peqGreen DNA/RNA dye (Peqlab, 37–5000).

#### LC-MS/MS

MS analysis was performed at the Proteomics Facility, University of Bristol. 50 μL of the samples (∼8–10 μg of protein) were run on a 4–12% NuPAGE Novex acrylamide gel (Life Sciences). The gel lane was cut into one slice and subjected to in-gel tryptic digestion using a ProGest automated digestion unit (Digilab UK). The resulting peptides were fractionated using a Dionex Ultimate 3000 nanoHPLC system in line with an LTQ-Orbitrap Velos mass spectrometer (Thermo Scientific). In brief, peptides in 1% (vol/vol) formic acid were injected onto an Acclaim PepMap C18 nano-trap column (Dionex). After washing with 0.5% (vol/vol) acetonitrile 0.1% (vol/vol) formic acid peptides were resolved on a 250 mm × 75 μm Acclaim PepMap C18 reverse phase analytical column (Dionex) over a 150 min organic gradient, using 7 gradient segments (1–6% solvent B over 1min, 6–15% B over 58 min, 15–32% B over 58min, 32–40% B over 3 min, 40–90% B over 1 min, held at 90% B for 6 min and then reduced to 1% B over 1 min) with a flow rate of 300 nL min^−1^. Solvent A was 0.1% formic acid and Solvent B was aqueous 80% acetonitrile in 0.1% formic acid. Peptides were ionized by nano-electrospray ionization at 2.1 kV using a stainless steel emitter with an internal diameter of 30 μm (Thermo Scientific) and a capillary temperature of 250°C. Tandem mass spectra were acquired using an LTQ- Orbitrap Velos mass spectrometer controlled by Xcalibur 2.1 software (Thermo Scientific) and operated in data-dependent acquisition mode. The Orbitrap was set to analyze the survey scans at 60,000 resolution (at m/z 400) in the mass range m/z 300 to 2,000 and the top twenty multiply charged ions in each duty cycle selected for MS/MS in the LTQ linear ion trap. Charge state filtering, where unassigned precursor ions were not selected for fragmentation and dynamic exclusion (repeat count, 1; repeat duration, 30s; exclusion list size, 500), was used. Fragmentation conditions in the LTQ were as follows: normalized collision energy, 40%; activation q, 0.25; activation time 10 ms; and minimum ion selection intensity, 500 counts.

#### Immunoblot analysis

0.05% of the yeast input extract (∼5 μg of protein) and 10%–50% of the eluates were resolved on 4–15% SDS polyacrylamide gels and transferred to polyvinylidene difluoride (PVDF) membranes (Thermo Scientific Pierce). Membranes were blocked in PBS-0.1% Tween 20 containing 5% low fat milk, probed with designated antibodies and horseradish peroxidase (HRP)-coupled secondary antibodies, and developed with the Immobilon Western Chemiluminescent HRP Substrate (Millipore). Blots were recorded with a FluorChem (Alpha Innotech) and quantified with ImageJ. The following antibodies were used: rabbit anti-Scp160 (Frey et al., 2001) (1:10,000), mouse anti-Act1 (1:2,500; MP Biomedicals, 0869100), rabbit anti-Pfk (Rodicio et al., 2000) (1:20,000), rabbit anti-Zwf1 (1:5,000, Sigma, A9521), mouse anti-Pgk1 (1:1,000; AbCam, ab113687, clone 22C5D8), PAP reagent (1:10,000, Sigma, P1291), HRP-conjugated donkey anti-rabbit IgG (1:5,000; Amersham, NA9340V), and HRP-conjugated sheep anti-mouse IgG (1:5,000; Amersham, NXA931). The validation of all commercial primary antibodies is provided on the manufacturer's website.

### Quantification and statistical analysis

#### Protein identification - TOP3 analysis

The raw data files were processed and quantified with the TOP3 method, which calculates the mean of the three highest peptide areas measured for each protein (Silva et al., 2006) using Proteome Discoverer software v1.2 (Thermo Scientific). Processed data was searched against the SwissProt SPECIES database using the Mascot algorithm (Version 2.4). Peptide precursor mass tolerance was set at 10 ppm, and MS/MS tolerance was set at 0.8 Da. Search criteria included carbamidomethylation of cysteine (+57.0214) as a fixed modification and oxidation of methionine (+15.9949) as a variable modification. Searches were performed with full tryptic digestion and a maximum of one missed cleavage was allowed. The reverse database search option was enabled, and all peptide data was filtered to satisfy a false discovery rate (FDR) threshold of <1%, obtaining 1,589 proteins. Only proteins represented with more than 2 peptides in at least one of the biological samples were considered for further analysis (1,070 proteins).

#### Protein identification - MaxQuant analysis

MaxQuant considers the sum of all peptide intensities divided by the number of observable peptides of a protein (Cox et al., 2014; Cox and Mann, 2008). LFQ is very similar to iBAQ but protein intensities are normalized and based on at least two ratio counts to exclude some ‘outliers’. All raw data were analyzed with MaxQuant software version 1.6.0.16 and mapped to the UniProt reference yeast database (downloaded 2017/05/23). MS/MS searches were performed with the following parameters: Oxidation of methionine and protein N-terminal acetylation as variable modifications; carbamidomethylation as fixed modification; Trypsin/P as the digestion enzyme allowing up to two missed cleavage sites; precursor ion mass tolerances of 20 p.p.m. for the first search (used for nonlinear mass re-calibration) and 4.5 p.p.m. for the main search, and a fragment ion mass tolerance of 20 p.p.m. For identification, we applied a maximum FDR of 1% separately on protein and peptide level. ‘Match between the runs’ was activated, as well as the ‘LFQ’ (at least two ratio counts were necessary to get an LFQ value), obtaining 1,585 proteins. We required 2 or more unique/razor peptides for protein identification and a ratio count of 2 or more for label free protein quantification in at least one of the 12 samples. This gave us LFQ/iBAQ values for a total of 1,310 protein groups.

#### Quantification and statistical analysis of MS data

Each of the three above described MS quantified datasets (TOP3, Maxquant in iBAQ and LFQ mode) were filtered to contain only the proteins identified with at least 2 peptides in at least one biological sample (control or H_2_O_2_ treated); 1,070 proteins with TOP3, and 1,310 proteins for both MaxQuant modes were retained. The filtered raw peak area data was normalized such that the total peak area within each biological sample was equal to 10^6^. A pseudocount of 1 was added to all normalized values and log_2_ transformed. Only proteins with an average (across replicates) raw biological peak area ≥ 3-fold greater than the corresponding average raw control peak area in both conditions were retained, leaving 935, 986 and 1,282 proteins in TOP3, LFQ and iBAQ analysis modes, respectively. Subsequently, proteins not identified in at least two of the three biological samples for at least one of the conditions were removed, leaving a final count of 812, 933 and 1,276 proteins in Top 3, LFQ and iBAQ analysis, respectively. These sets of proteins were further analyzed to estimate an FDR for declaring differentially abundant proteins between conditions. Since samples were not paired an average pairwise fold-change between replicate samples was calculated to identify proteins that change in abundance/presence between conditions. The probability of observing such a fold-change by chance (i.e. FDR) (which informed whether to declare a protein as being differentially abundant between conditions), was based on the distribution of average fold-changes generated from 10,000 random experiments. Random experiments were generated by shuffling the experimentally obtained fold-changes of replicates. From this randomized dataset, the average log_2_ fold-changes were calculated to assess, for each possible log_2_ H_2_O_2_/WT threshold of −10 to 10 in 0.5 increments, the percentage of the 10,000 randomly generated fold-changes that were above that threshold i.e. the FDR. From this simulated data a linear model of the form FDR ∼ log_2_ H_2_O_2_/WT fold-change was created and subsequently used to produce FDRs for all experimentally obtained (non-random) fold changes. Proteins having an FDR ≤5% were considered to be differentially abundant (Pairwise comparison and FDR values are given in Tables S1A–S1C for TOP3, LFQ and iBAQ analysis, respectively).

#### Database searches

Significantly enriched GO terms in *S. cerevisiae* (p < 0.01, FDR <5%) were identified with the GO Term Finder at the *Saccharomyces* Genome Database (Costanzo et al., 2014) (SGD; http://www.yeastgenome.org/) (complete analysis is given in Table S2A). Pathway analysis (Wikipathway, KEGG, Reactome, and Panther) was conducted with WebGestalt (Liao et al., 2019), considering all protein coding genes as the background set (6,725 genes) (Table S2B). GSEA was performed with Webgestalt, considering the average FC ranked data of the entire mRBPome (Table S2C). Of note, Webgestalt uses the p.adjust function of R to correct for multiple testing and thus, the adjusted p values reported have the same interpretation as the GO enrichment analyses i.e. all adjusted p values have an FDR <5%. Two genes were selected as the minimum number for a category.

#### Venn diagram p values

Overlap significance of Venn diagrams was calculated using hypergeometric distributions.

## Supplemental information

Document S1. Figures S1–S3

Table S1. Normalized MS data for S. cerevisiae samples defining changes of the mRBPome upon oxidative stress, related to Figure 1 Statistical analysis of normalized MS data (see STAR Methods) is shown in panels A-C. The normalized MS data for each biological sample (WT1-3, H_2_O_2_1-3), average pairwise fold-change, and FDRs (%) are provided in different columns. Proteins that changed RNA association significantly (FDR ≤ 5%) in H_2_O_2_ treated cells are marked with the filled yellow box (FDRs). (A) “TOP3” analysis. mRBPome specifies the 812 proteins depicted in Figure 1B. Thereby, “All” refers to proteins identified both, in 2 of 3 untreated and H_2_O_2_ treated samples; “Untreated” refers to proteins exclusively identified in 2 of 3 untreated samples, “H_2_O_2_”, proteins identified in 2 of 3 stressed samples. (B) MaxQuant Label-Free Quantification (LFQ). (C) MaxQuant Intensity-Based Absolute Quantification (iBAQ). (D) List of protein overlap described in a Venn diagram show in Figure S2C. (E) The 257 proteins that significantly changed poly(A) RNA associations (FDR ≤ 5%; Figure 1D) in H_2_O_2_ treated cells are highlighted in yellow and depicted within a list of all proteins identified at 1% FDR and at least 2 peptides in two of the three biological MS replicates (total 1,341 proteins). Different columns refer to average log_2_ FC (H_2_O_2_/wt) across the three MS analysis methods and their average.

Table S2. Analysis of the proteins differentially associated with mRNAs upon oxidative stress, related to Table 1 (A) Significantly enriched GO terms for Biological Process, Molecular Function, and Cellular Component (p < 0.01) among the 107 proteins with significantly decreased (blue), the 150 proteins with significantly increased (yellow), and all the 257 (gray) with significantly changed poly(A) RNA association upon oxidative stress. Columns indicate the following (from left to right): GO Category: GO Biological Process/ Molecular Function/ Cellular Component; Set of proteins for enrichment analysis; GO Identity; GO term; Cluster frequency, the number of genes of the corresponding subgroup of proteins assigned to the term; Background frequency, the number of ORFs annotated with the term; p-value; FDR; and Gene(s) associated with the term. (B) Enriched pathways (p < 0.05) among the set of proteins that significantly changed poly(A) RNA associations in H_2_O_2_ treated cells. Columns indicate the following (from left to right): Category, refers to the database selected for pathway enrichment analysis; Set of proteins; Gene Set; Description; Size, the number of genes allocated to the term in the background gene list; Expect, number of proteins expected to be retrieve by chance; Ratio, value over expected; No proteins, number of proteins in respective set of proteins allocated to the indicated pathway; p-value; FDR. (C) GSEA (p < 0.01) performed with the ranked list of 1,341 proteins (Table S1E) identified at 1% FDR with at least 2 peptides in two out of the three biological MS replicates from untreated and/or H_2_O_2_ treated cells.

Table S3. List of paralogous proteins interacting with RNA, related to Figure 2 The list contains 53 paralogous pairs and 91 proteins identified irrespective of its paired paralogue among the 812 proteins defined as mRBPome upon oxidative stress with TOP3 method.
