# Supplementary material for: Oxidative stress induces coordinated remodeling of RNA-enzyme interactions
Source: iScience. 2021 Jun 19;24(7):102753. doi: 10.1016/j.isci.2021.102753 (PMC8261671; doi:10.1016/j.isci.2021.102753)
Supplement: Document S1. Figures S1–S3 [file mmc1.pdf]

iScience, Volume *24*

Supplemental information

Oxidative stress induces coordinated remodeling of RNA-enzyme interactions

Ana M. Matia-González, Ibtissam Jabre, Emma E. Laing, and André P. Gerber

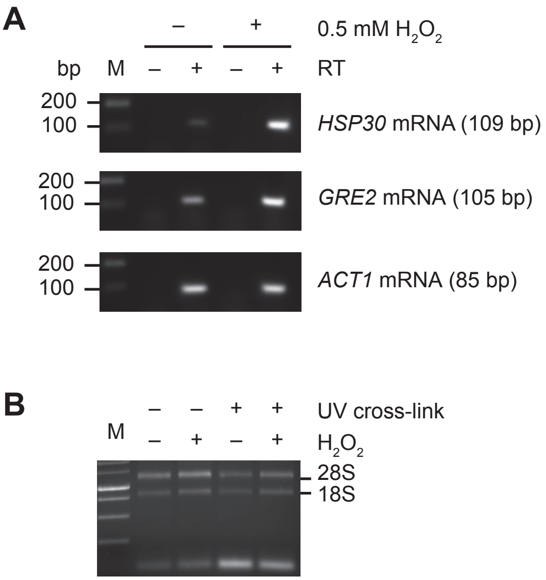

Figure S1. Validation of oxidative stress response and RNA integrity. Related to Figure 1.
(A) RT-PCR on total RNA isolated from untreated and 0.5 mM H_2O_2 treated yeast cells using *HSP30*, *GRE2* and *ACT1* specific primers. Products were visualized on an agarose gel. Increased *HSP30* and *GRE2* mRNAs levels are a marker for oxidative stress response, while *ACT1* mRNA levels are not expected to change and served as a negative control.
(B) One µg of total RNA from untreated and 0.5 mM H_2O_2 treated cells was electrophoresed on a 1% agarose gel and visualized with Red-safe. Total RNA from non-irradiated cells was used as a reference.

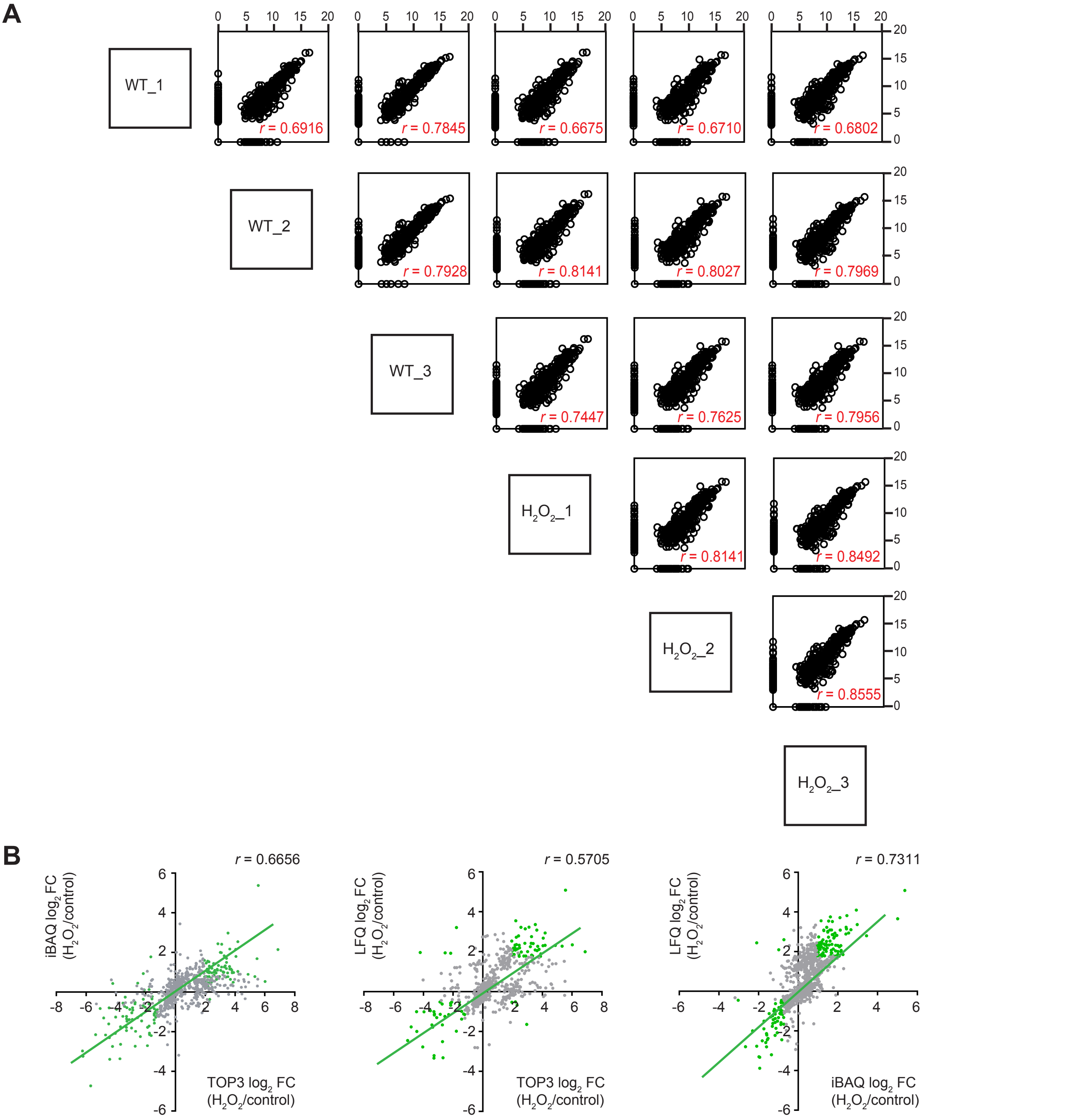

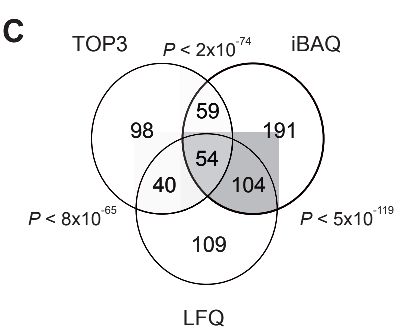

Figure S2. Comparison of *S. cerevisiae* mRBPome MS samples and label-free quantification methods. Related to Figure 1 and Table S1.

(A) Scatter plots comparing the processed (see STAR Methods) protein peak areas between all yeast samples defined with the TOP3 method. The samples being directly compared within a plot can be identified by the 'label' boxes along the diagonal, where the sample plotted on the *y*-axis is identified by the label box to the left of the scatterplot and the sample on the *x*-axis is identified by the label box beneath the scatterplot. The respective Pearson correlation coefficient (*r*) , calculated using the processed peak areas, of a comparison is indicated to the bottom of each scatterplot.

(B) Scatter plots representing the overlap of the log_2 FC (H_2O_2/WT) of proteins selected with TOP3 (812 proteins), iBAQ (1,276; left panel) or LFQ (933; central panel), and iBAQ and LFQ (right panel). Proteins with FDR ≤ 5% in iBAQ or LFQ analysis are highlighted in green. The Pearson correlation (*r*) values between TOP3 and iBAQ, TOP3 and LFQ, and iBAQ and LFQ are indicated at the top right corner.

(C) Venn diagram representing the overlap of proteins differentially associated with poly(A) RNAs and selected by the indicated MS analysis method with FDR ≤ 5%. *P*-values were calculated using hypergeometric distribution.

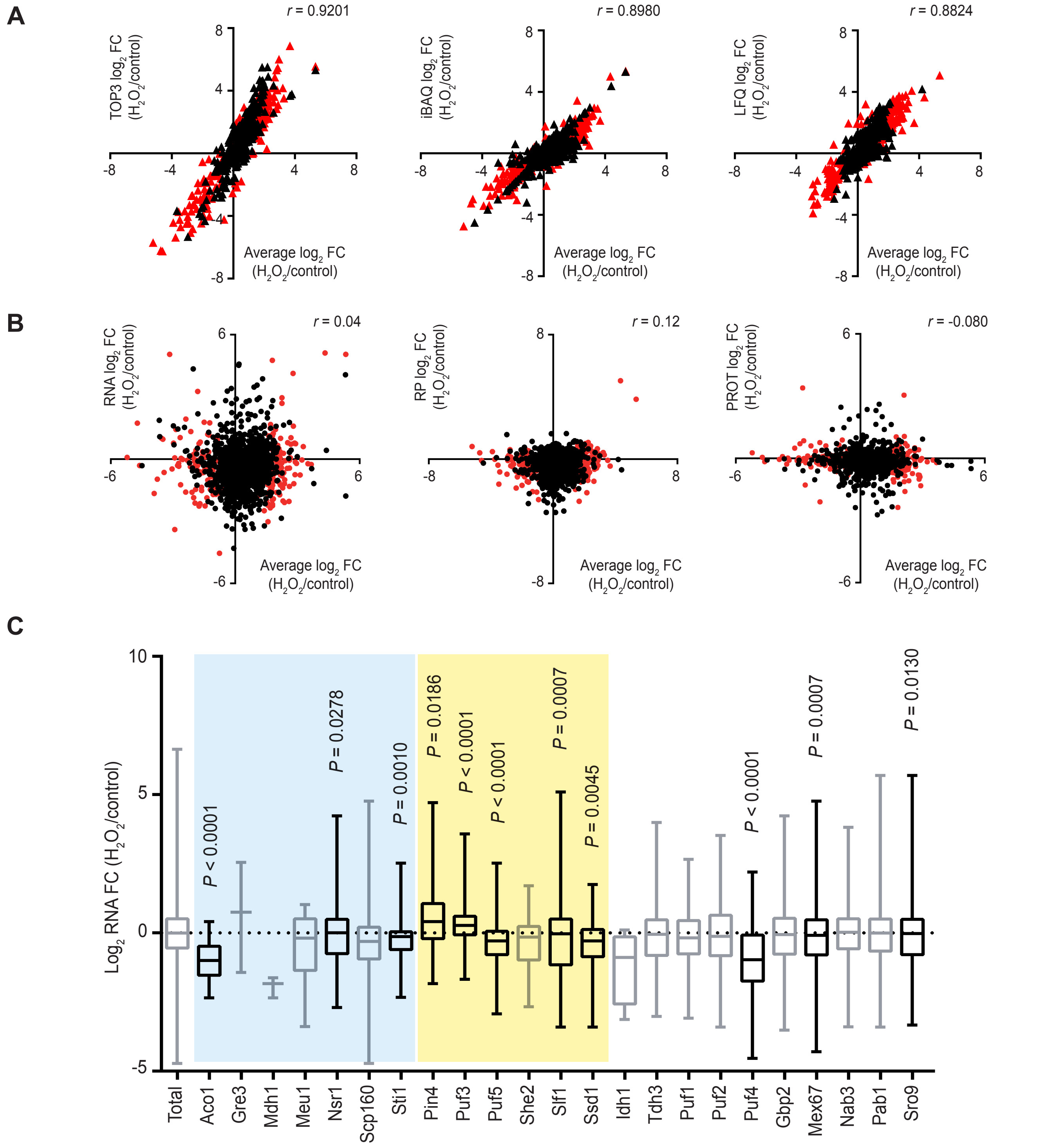

Figure S3. Comparison of mRNA levels, ribosome occupancies and protein levels of RBPs upon oxidative stress conditions. Related to Figure 1.

(A) Scatter plots depicting average $\log_2$ FC (H_2O_2/control) of individual proteins (triangles) obtained from three MS quantification methods (*x*-axis) and respective value from the three quantitative methods individually (*y*-axis). The Pearson correlation coefficient (*r*) is indicated to the top right of each scatter plot. The 257 proteins selected as differential binders of poly(A) RNA upon stress are highlighted in red.

(B) Scatter plots depicting a comparison between the average $\log_2$ FC (H_2O_2/control) values obtained across all quantitative methods for mRBPome and the average $\log_2$ FC (H_2O_2/control) of RNA levels after treatment of cells with 0.4 mM H_2O_2 for 20 min (Alejandro-Osorio *et al*., 2009) (left panel); changes in ribosome occupancy upon treatment of cells with 0.2 mM H_2O_2 for 30 min (Gerashchenko *et al*., 2012) (middle panel); and changes in the protein levels (Blevins *et al*., 2019) (right panel) upon application of 1.5 mM H_2O_2 for one hour. The Pearson correlation coefficient (*r*) is indicated at the top; the 257 proteins selected as differential binders of poly(A) RNA upon stress are highlighted in red.

(C) Relative changes in expression levels for published RNA targets of 23 proteins (Hieronymus and Silver, 2003; Hogan *et al*., 2008; Schenk *et al*., 2012; Scherrer *et al*., 2010) upon application of 0.4 mM hydrogen peroxide for 20 min. Boxplot depicting relative changes of mRNA levels of H_2O_2 treated compared to untreated cells (Alejandro-Osorio *et al*., 2009) (*y*-axis) for RNA targets of the indicated RBPs (*x*-axis). RBPs within the blue box denote RBPs with reduced RNA associations upon H_2O_2 treatment; the yellow box highlights RBPs with increased associations. Filled boxes extend from the first to the third quartile whilst whiskers extend to minimum and maximum values. *P*-values were determined in a Mann-Whitney two-tailed test comparing the distribution of RBPs' targets to total RNA expression.
